# Smartphone‐Supported Cognitive‐Behavioral Therapy in Binge‐Eating Disorder: An Exploratory Randomized Trial

**DOI:** 10.1002/eat.24479

**Published:** 2025-06-16

**Authors:** Anja Hilbert, Ulrike Klotz, Sina Sadeghi, Adrienne S. Juarascio, Toralf Kirsten

**Affiliations:** ^1^ Integrated Research and Treatment Center Adiposity Diseases, Behavioral Medicine Research Unit, Department of Psychosomatic Medicine and Psychotherapy Leipzig University Medical Leipzig Germany; ^2^ Institute for Medical Informatics, Statistics and Epidemiology Leipzig University Leipzig Germany; ^3^ Department for Medical Data Science Leipzig University Medical Center Leipzig Germany; ^4^ Department of Psychological and Brain Sciences; Weight, Eating, and Lifestyle Sciences Center Drexel University Philadelphia USA

**Keywords:** adaptive intervention, binge‐eating disorder, cognitive‐behavioral therapy, ecological momentary intervention, just‐in‐time adaptive intervention, mHealth, mobile health, smartphone application

## Abstract

**Objective:**

To assess the feasibility of a smartphone app delivering just‐in‐time adaptive interventions as an adjunct to cognitive‐behavioral therapy (CBT) adapted to binge‐eating disorder (BED), estimate its effects assuming superiority over CBT alone, and document safety and target engagement.

**Method:**

A single‐center, assessor‐blinded, parallel feasibility study randomized adults aged 18–65 years with full‐syndrome or subthreshold BED to smartphone‐supported CBT (SmartCBT) or standard CBT (DRKS00024597). Both arms received 16 individual 50‐min CBT sessions over 4 months. Assessments were conducted at baseline (T0), midtreatment (T1), posttreatment (T2), and 3‐month follow‐up (T3). Feasibility was determined regarding recruitment, attrition, dropout, adherence, assessment completion, app use, and acceptance. Further, eating disorder symptoms, mental and physical health, weight management behavior, safety, and target engagement (i.e., skill use) were assessed.

**Results:**

Over a 7‐month recruitment period, 28 of 50 eligible volunteers were included and randomized 1:1 to SmartCBT or CBT. In the modified intent‐to‐treat sample (*N* = 25; SmartCBT: 13, CBT: 12), the feasibility of SmartCBT was further supported regarding attrition, dropout, adherence, treatment completion, app use, and acceptance; however, assessment completion was moderate. Clinical improvements were found in both arms, but differential results were affected by baseline differences and moderate assessment completion in the SmartCBT arm. Safety was documented, and support for target engagement was found.

**Conclusions:**

This exploratory study provides evidence for the feasibility of app‐supported CBT for BED. With few procedural refinements, the protocol can be used in a confirmatory randomized‐controlled trial with long‐term follow‐up to evaluate efficacy and determine treatment mechanisms.

**Trial Registration:**

German Clinical Trials Register, https://www.drks.de, DRKS00024597

**Summary**

- Cognitive‐behavioral therapy (CBT) for binge‐eating disorder (BED) is well‐established, but patients often struggle to apply therapy‐taught skills in daily life.
- A smartphone application, adapted for BED to provide personalized support for daily skill use, was piloted in a randomized trial comparing smartphone‐supported CBT with standard CBT.
- The results offer initial evidence on the feasibility of study procedures, the app's skills‐focused support in CBT, and utility and safety for patients.

## 1 Introduction

Binge‐eating disorder (BED) is the most prevalent clinical eating disorder, characterized by recurrent episodes of binge eating without regular weight‐compensatory behaviors (American Psychiatric Association [APA] 2013). Affecting 1.5% of women and 0.3% of men globally (Santomauro et al. 2021), BED is associated with elevated eating disorder and general psychopathology, obesity (BMI ≥ 30.0 kg/m^2^), comorbidity (e.g., cardiometabolic diseases), and impaired quality of life (Giel et al. 2022). Cognitive‐behavioral therapy (CBT) is the most established treatment for BED, yielding sustained reductions in binge eating and associated psychopathology and stabilizing body weight, though full remission is achieved in only ~50% of cases (Hilbert et al. 2019). Treatment success in CBT hinges on the use of specific skills (Fairburn 2008), with insufficient skill use linked to poorer outcomes (Srivastava et al. 2023). Therefore, supporting patients in applying CBT skills, particularly in challenging real‐life situations, is crucial for optimal outcomes (Leehr et al. 2018).

Mobile health (mHealth) systems that provide personalized support for skill use at critical moments in daily life may effectively address this treatment gap. Although many electronic health interventions, including mHealth technologies, have been developed (Anastasiadou et al. 2018; Barakat et al. 2019), few are evidence‐based (O'Leary and Torous 2022). While most research focuses on feasibility (Anastasiadou et al. 2020), several larger‐scale randomized controlled trials (RCTs) report initial efficacy of smartphone apps for eating disturbances, including BED, as stand‐alone or blended treatments (Linardon et al. 2022, 2023; Hildebrandt et al. 2020). However, their capacity to augment standard care remains unclear, especially regarding therapist‐guided, personalized apps (Tregarthen et al. 2019).

Ecological momentary interventions (EMIs) offer a framework for personalized interventions by providing real‐time support in challenging daily life situations based on ecological momentary assessment data (Nahum‐Shani et al. 2012, 2015, 2018). Adaptive, personalized EMIs, or just‐in‐time adaptive interventions (JITAIs), have shown promise for various health behaviors (Hsu et al. 2025; Wang and Miller 2020). Among smartphone apps for eating disorders (Ahmadiankalati et al. 2020; Dufour et al. 2022), only one, CBT+, has empirical support for delivering JITAIs. Initially developed for bulimia nervosa, CBT+ supports skill use between sessions to normalize eating behavior and improve emotion and impulse regulation—core maintenance factors in CBT for binge eating (Fairburn 2008; Hilbert and Tuschen‐Caffier 2010). Feasibility and acceptability were supported in a pilot RCT (*N* = 56), with large improvements in skill use and clinically significant reductions in binge‐purge symptoms in app‐supported CBT, both with and without the provision of JITAIs (Juarascio et al. 2021, 2023). However, it is unknown whether CBT+ is feasible for BED, especially considering necessary adaptations for this disorder's clinical profile.

This exploratory RCT aimed to evaluate the feasibility and estimate the effects of CBT with versus without an adjunctive CBT+ app adapted for BED. It was hypothesized that CBT with the adjunctive smartphone app (SmartCBT) would be feasible in terms of recruitment, attrition, treatment dropout, adherence to sessions, assessment completion, app use, patient perceived utility, usability, and satisfaction, and to yield superior efficacy estimates compared to CBT alone. Further aims included exploring safety and target engagement (i.e., therapeutic skill use).

## 2 Method

### 2.1 Study Design and Procedure

**FIGURE 1:**
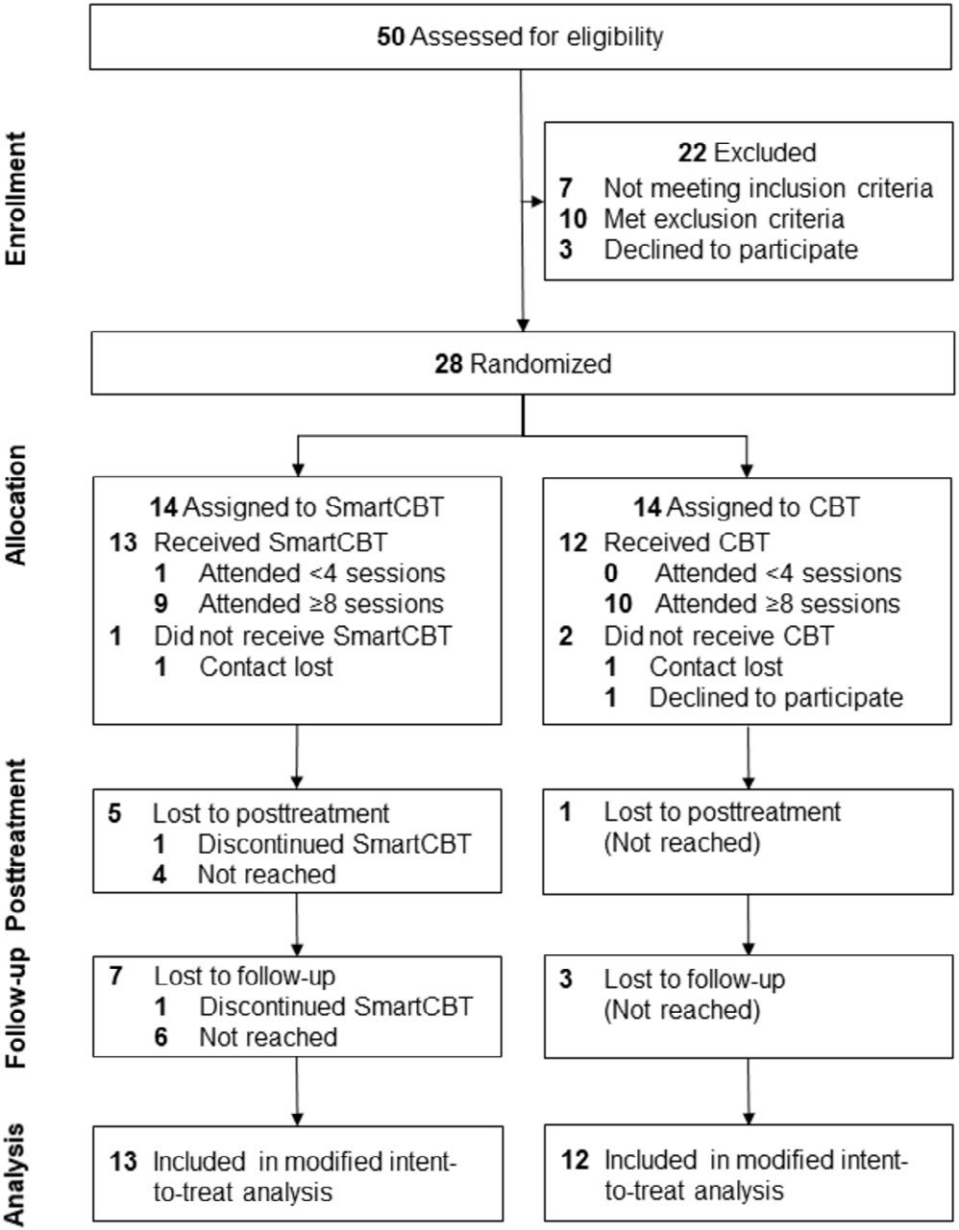
CONSORT flow diagram. Displayed is the patient flow from enrollment to follow‐up, specifying attrition (treatment discontinued or not received: 2/14 per arm, 4/28 total) and from those receiving treatment, early treatment dropout (attendance of < 4 sessions, SmartCBT: 1/13, CBT: 0/12), treatment completion (attendance of ≥ 8 sessions, SmartCBT: 9/13, CBT: 10/12), and assessment completion of the primary efficacy estimate at posttreatment (SmartCBT: 8/13, CBT: 11/12) and follow‐up (SmartCBT: 6/13, CBT: 9/12). CBT, cognitive‐behavioral therapy; SmartCBT, smartphone‐supported CBT.

The SmartCBT study is a single‐center, assessor‐blinded feasibility RCT, randomizing patients to SmartCBT or CBT. Ethical approval was obtained from the Leipzig University Ethical Committee (110/20‐ek), and the study was registered in the German Clinical Trials Register (https://www.drks.de; DRKS00024597). Written informed consent was obtained from all participants. Recruitment occurred via advertising and clinical referrals at Leipzig University Medical Center (Figure 1), with compensation of €100 for posttreatment and follow‐up assessments. Eligible participants were adults aged 18–65 years with a diagnosis of BED or BED of low frequency and/or limited duration according to the Diagnostic and Statistical Manual of Mental Disorders 5th edition (DSM‐5; APA 2013); a smartphone (Android) with Internet access; feasible commute; sufficient German language skills; and informed consent. Exclusion criteria were serious mental or physical illness; medical or psychological treatment impacting eating behavior; previous or planned bariatric surgery; and pregnancy or lactation.

### 2.2 Control: CBT

The treatment‐as‐usual control condition consisted of 16 individual 50‐min sessions of manualized, modular CBT over 4 months (Hilbert and Tuschen‐Caffier 2010), the standard of care for BED in Germany (Herpertz et al. 2020), sufficient for clinically significant change. CBT focuses on normalizing eating behavior, fostering a positive body image, and improving stress and emotion regulation, with established efficacy and safety (de Zwaan et al. 2017; Hilbert et al. 2019). CBT was delivered by MSc‐ or PhD‐level psychologists trained in CBT who received study‐specific training in CBT for BED and ongoing monthly supervision.

### 2.3 Intervention: SmartCBT

The CBT+ system, consisting of the CBT+ app and a web‐based clinician portal developed for bulimia nervosa (Juarascio et al. 2021, 2023), was chosen for its EMI design, personalized JITAIs, clinician‐guided tailoring, cognitive‐behavioral foundation, mechanism‐oriented operationalization, and scalability. For the exploratory SmartCBT RCT, we translated the CBT+ texts into German, shortened them to ensure feasibility while keeping app functionality the same. Further, we adapted intervention texts to BED and incorporated new app support for skills regarding eating behavior and body image to comprehensively address the maintenance of BED, including information, JITAIs, automated reminders, therapist‐defined push notifications, and self‐monitoring (Table 1). Specifically, considering BED's link to overeating and obesity (Heaner and Walsh 2013; Masheb et al. 2011), an adapted skill addressing balanced, calorie‐restricted eating was included. This skill is derived from CBT for BED (Hilbert and Tuschen‐Caffier 2010; de Zwaan et al. 2017) and promotes flexible, restrained eating through precise dietary self‐monitoring, using the German Nutrient Data Base (Bundeslebensmittelschlüssel 3.02; https://blsdb.de/). Nutritional information was provided per food, meal, and day to support normalization of eating patterns and weight stabilization, rather than weight reduction. Of note, CBT for BED was found to lead to an overall decrease in dietary restraint across sessions (Schmidt et al. 2025). Because of hedonic overeating and binge eating in BED (Schaefer et al. 2023), a new skill was specified helping patients to allow enjoyment of regular food intake. Skills to foster a positive body image were included because of the relevance of a negative body image in the maintenance of BED (Lewer et al. 2017): App support was delineated to help patients identify negative body‐related thoughts, feelings, and behaviors; develop positive body‐related behavior and an active lifestyle; and encourage healthy weight maintenance. Therefore, we included self‐monitoring of body image, physical activity, and weight. We retained the regular eating skill and all emotion and impulse regulation skills from the CBT+ app for bulimia nervosa, applicable to BED (Dingemans et al. 2017; Kittel et al. 2015; Schaefer et al. 2023). Unlike the original CBT+ app, which operates on iOS, we developed it for Android, the most widely used operating system in Germany (https://de.statista.com). The modified app was renamed trEATsmart.

**TABLE 1:** Cognitive‐behavioral therapy goals and therapeutic targets and modules of the trEATsmart app.

Therapeutic goals	Therapeutic skills/trEATsmart targets	trEATsmart modules	Target engagement assessed in trEATsmart
Normalization of eating behavior	Plan and realize regular eating	Regular eating	*n* intervals > 1 h and < 4 h between eating episodes per day with data entry per period when the regular eating module was activated
	Plan and realize a balanced, calorie‐moderated diet^a^	Balanced eating	kcal intake per day with data entry per period when the balanced eating module was activated
	Allow enjoyment of food^b^	Allow enjoyment	‐^c^
Emotion and impulse regulation	Identify triggers of urge to eat/craving	Trigger identification	‐^c^
	Use emotion regulation to reduce negative affect	Emotion regulation	*n* emotion regulation skills used during periods when the emotion regulation module was activated
	Tolerate urges to eat/craving without giving in	Impulse regulation	*n* impulse regulation skills used during periods when the impulse regulation module was activated
Develop a positive body image^b^	Identify negative body‐related self‐talk^b^	Positive body image	*n* self‐monitoring records of body image during periods when the positive body image module was activated
	Plan and realize regular physical activity^b^	Regular physical activity	*n* self‐monitoring records of physical activity during periods when the regular physical activity module was activated
	Plan and realize regular self‐weighing^b^	Regular weighing	*n* self‐monitoring records of body weight during periods when the regular weighing module was activated

*Note*: From Juarascio et al. (2021, 2023).

^a^ Adapted.

^b^ Newly developed for BED.

^c^ No app‐based assessment available.

Procedurally, the trEATsmart app facilitates electronic self‐monitoring between therapeutic sessions, aligned with collaboratively set therapeutic goals. Based on self‐monitoring and activated “priority goals,” an embedded algorithm determines whether to deliver a JITAI. These JITAIs, selected via multilevel decision trees, address specific barriers to skill use (e.g., lack of coping strategies), provide rationale and guidance, and encourage immediate behavioral adjustments, referring to the therapy sessions (for detail, see Juarascio et al. 2021). To prevent overload, only current priority goals are targeted weekly. Patients are encouraged to indicate intended use of JITAI‐suggested strategies, rate weekly goal achievement, and flag entries for in‐session discussion. A complementary skill repository provides access to therapy‐related content at any time.

The web‐based clinician portal allows therapists to monitor patients' app use, self‐monitoring, and goal achievement, assign weekly goals, and adjust interventions. Priority goals typically evolve over therapy (e.g., from eating behavior and mood to body image), while earlier goals may remain active to consolidate skills. Therapists can select or personalize goals, skills, and JITAIs, define personalized push notification interventions (e.g., self‐weighing reminders at a specific time and day), and customize automated reminders (e.g., prompts for data entry with inactivity ≥ 6 waking hours). The portal provides summary or detailed self‐monitoring data for therapeutic work during sessions and to activate new goals and skills post‐session. Thus, therapist time in SmartCBT and CBT sessions was equivalent. JITAI delivery and patient responses were tracked to identify barriers and support refinement.

Technically, the app, downloadable on patients' smartphones, operates without an Internet connection. Data were stored anonymously on a secure, encrypted server in Germany and downloaded behind the firewall of Leipzig University Medical Center. All communication with the app was encrypted.

trEATsmart was integrated into 16 individual 50‐min sessions of manualized CBT over 4 months as described for the control condition. Therapists delivered treatment in both arms under monthly supervision by AH. In addition, they received training in the app's use in CBT by ASJ, and a therapist and patient manual for the app (Juarascio et al. 2021, 2023) was adapted for this study.

An initial pilot study (*N* = 5) tested the app with iterative refinements, following the same procedures as in the RCT (data not reported here).

### 2.4 Randomization and Sample Size Estimation

After baseline assessment, patients were randomized by computer in variable‐length blocks (2 or 4 patients), stratified by sex (female, male), with a 1:1 allocation ratio. Given this study's exploratory nature, sample size was based on parameter precision, not expected incremental effects of the app. A sample of 20 patients (10 per arm) was deemed sufficient to estimate OBEs with a sufficiently narrow confidence interval (CI) for planning a subsequent confirmatory RCT. This sample size calculation was based on a meta‐analytically determined increase in effectiveness of mHealth technologies in psychotherapy by *d* = 0.27 (Lindhiem et al. 2015). Using this information, the 80% CI for planning the follow‐up study would have a width of approximately 1.1 standard deviations. With a meta‐analytically determined dropout rate of 19% in psychotherapy for BED (Hilbert et al. 2019), 24 patients were sought to be recruited for the study.

### 2.5 Measures

Assessments were conducted at baseline (T0), midtreatment after 2 months (T1), posttreatment after 4 months (T2), and at the 3‐month follow‐up after the end of treatment (T3). Feasibility measures included recruitment (i.e., ≥ 2/month to achieve the target sample size within a 12‐month recruitment period), attrition (≤ 20% treatment discontinued or not received; Hilbert et al. 2019), early treatment dropout (attendance of < 4 sessions, i.e., < 25%), treatment completion (attendance of ≥ 8 sessions, i.e., ≥ 50%; Linardon et al. 2017), adherence to sessions (*n* sessions attended), and assessment completion of the primary efficacy estimate at T2 (0 = noncompletion, 1 = completion).

App use was tracked via trEATsmart logs, providing the following indicators: *n* app use per day; n entries per day; *n* JITAIs delivered; percentage of these JITAIs planned to be implemented by patients; percentage of patients using app‐based self‐monitoring of eating, physical activity, weight, and body image. Patients rated utility by module and usability of the app (SmartCBT only), and overall satisfaction with treatment (all patients) in terms of perceived and expected treatment success at T2 (0 = not at all to 10 = extremely). In addition, problems with the app were rated at T2 as 1 = not at all to 7 = extremely.

Regarding efficacy estimates, the primary estimate was the number of objective binge‐eating episodes (OBEs), defined as consuming large amounts of food with a sense of loss of control over eating, in the past 28 days at T2, assessed with the Eating Disorder Examination (EDE; Fairburn et al. 2014; Hilbert and Tuschen‐Caffier 2016), a semi‐structured interview with established reliability and validity (Berg et al. 2012). Secondary estimates included the number of OBEs at T3, abstinence from binge eating (i.e., zero OBEs over the past 28 days), and remission from BED (including BED of low frequency and/or limited duration) per DSM‐5 criteria (APA 2013), all assessed using the EDE at T2 and T3. Secondary measures included eating disorder psychopathology (Eating Disorder Examination‐Questionnaire, EDE‐Q; Fairburn and Beglin 2008; Hilbert and Tuschen‐Caffier 2016); anxiety disorder symptoms (Generalized Anxiety Disorder 7, GAD‐7; Löwe et al. 2008; Spitzer et al. 2006); global self‐efficacy (General Self‐Efficacy Scale, GSES; Schwarzer and Jerusalem 1995); emotion regulation deficits (Difficulties in Emotion Regulation Scale; DERS; Ehring et al. 2008; Gratz and Roemer 2004); impairment due to eating disorder psychopathology (Clinical Impairment Assessment, CIA; Bohn et al. 2008; de Zwaan et al. 2017); and weight‐related quality of life (Impact of Weight on Quality of Life–Lite, IWQOL‐Lite; Kolotkin et al. 2001; Mueller et al. 2011; see Supporting Information for details). BMI (kg/m^2^) and waist‐to‐hip ratio (WHR) were determined from objective measurements.

Safety was assessed by therapists before each session and by self‐report via the Patient Health Questionnaire (PHQ‐15; Gräfe et al. 2004; Spitzer et al. 1999). Target engagement was measured via trEATsmart logs (cf. D'Adamo et al. 2024; Table 1).

### 2.6 Data Management and Analysis

Study data were released for analysis after study completion only, and interim analyses were not conducted. The follow‐up period ended on 07/2022, and data were analyzed from 03/2023 to 09/2024.

Analyses were conducted on the modified intent‐to‐treat sample, including all randomized participants who attended ≥ 1 treatment session, to reduce attrition bias in estimating treatment effects by study arm. Meta‐analytically, the modified intent‐to‐treat approach was found to produce estimates comparable to the intent‐to‐treat approach (Dossing et al. 2016). All data were used without prior imputation because the small sample size made meaningful multiple imputation of missing values unfeasible.

Feasibility, app use, treatment evaluation, and safety were reported descriptively and/or compared by arms using standardized effect sizes with 95% CIs. Regarding the efficacy estimates, the primary analysis compared the change in the number of OBEs T0–T2 by treatment arm using standardized effect sizes with 95% CIs. In addition, for all efficacy estimates, linear mixed models were conducted by arm, with gender as a fixed effect, time treated as a categorical variable (T0, T1, T2, T3), and patients treated as a random term; adjusted effects and effect sizes (Cohen’s *d*; Cohen 1988) with 95% CIs were reported. Categorical estimates were analyzed using number needed to treat (NNT) with 95% CIs (Kraemer and Kupfer 2006). Target engagement in the SmartCBT arm was reported descriptively and Pearson's *r* was calculated with T2 OBE improvement. All analyses were conducted with R (version 4.1.1), in particular using *lme4* and *effsize*.

## 3 Results

### 3.1 Participants

Recruitment took place between 06 and 12/2021. Of a total of 50 volunteers screened for eligibility over the telephone, *N* = 28 patients met the inclusion criteria, determined by in‐person assessment (Figure 1). Patients were randomized to SmartCBT or CBT (*n* = 14 each), with *n* = 25 patients having obtained ≥ 1 treatment session retained for the modified intent‐to‐treat sample (SmartCBT: 13, CBT: 12).

As displayed in Table 2, most patients were female, middle‐aged, had low school education, and obesity. According to DSM‐5, 92% of the patients were diagnosed with BED, while 17% of patients, all assigned to the CBT arm, had BED of low frequency and/or limited duration. Descriptively, the most notable sociodemographic or clinical differences were approximately 20% higher rates of patients with low school education and with anxiety disorders in the SmartCBT than in the CBT arm. Regarding patients' reasons for study participation assessed prior to randomization, the reasons for study participation were likely to be multifold for SmartCBT patients but mostly related to weight loss in CBT patients. Patients perceived the suitability of and their motivation for treatment to be high or very high, while their belief in treatment success was moderate.

**TABLE 2:** Baseline sociodemographic characteristics and motivation.

	SmartCBT	CBT	Total
	(*n* = 13)	(*n* = 12)	(*N* = 25)
	*M* (SD)/*n* (%)	*M* (SD)/*n* (%)	*M* (SD)/*n* (%)
Sex, female	9 (69%)	7 (58%)	16 (64%)
Age, years	42.5 (12.5)	39.2 (13.2)	40.9 (12.7)
Education			
≥ 12 years	4 (31%)	6 (50%)	10 (40%)
< 12 years	9 (69%)	6 (50%)	15 (60%)
Body mass index, kg/m^2^	39.1 (9.0)	41.6 (8.7)	40.3 (8.8)
Weight status	113.3 (30.5)	123.2 (29.5)	118.1 (29.8)
Normal weight, 18.5–24.9 kg/m^2^	2 (15%)	0	2 (8%)
Overweight, 25.0–29.9 kg/m^2^	1 (8%)	1 (8%)	2 (8%)
Obesity class 1, 30.0–34.9 kg/m^2^	0	1 (8%)	1 (4%)
Obesity class 2, 35.0–39.9 kg/m^2^	2 (31%)	3 (25%)	5 (20%)
Obesity class 3, ≥ 40.0 kg/m^2^	8 (46%)	7 (50%)	15 (60%)
Eating disorder diagnosis (DSM‐5)^a^			
BED	13 (100%)	10 (83%)	23 (92%)
BED of low frequency and/or limited duration	0	2 (17%)	2 (8%)
Mental comorbidity			
Major depression (PHQ‐D ≥ 10)	6 (46%)	5 (42%)	11 (44%)
Generalized anxiety disorder (GAD‐7 ≥ 15)	6 (46%)	3 (25%)	9 (36%)
Reasons for study participation^b^			
Binge‐eating improvement	3 (23%)	3 (25%)	6 (24%)
Weight loss	2 (15%)	7 (58%)	9 (36%)
Interest in smartphone‐supported therapy	1 (8%)	0	1 (4%)
Multiple reasons	6 (50%)	2 (29%)	8 (32%)
Therapy expectations (0–10)			
Suitability	7.6 (1.8)	8.6 (1.7)	8.1 (1.8)
Success	6.9 (1.2)	7.9 (1.5)	7.4 (1.4)
Motivation	8.9 (1.6)	9.6 (0.7)	9.2 (1.2)

*Note*: BED, binge‐eating disorder; BMI, body mass index (kg/m^2^), derived from measured height and weight; CBT, cognitive‐behavioral therapy; DSM‐5, Diagnostic and Statistical Manual of Mental Disorders, Fifth Edition; GAD‐7, Generalized Anxiety Disorder 7‐Item Scale; PHQ‐D, Patient Health Questionnaire‐Depression Scale; SmartCBT, smartphone‐supported CBT.

^a^ Eating disorder diagnosis determined through the Eating Disorder Examination.

^b^ Data missing from one patient (SmartCBT).

### 3.2 Feasibility

Feasibility was documented including timely recruitment within 7 months with 4.4 patients/month, thus about twice as fast as planned (2 patients/month over 12 months). As can be seen in Figure 1, attrition (i.e., treatment discontinued or not received) was low, occurring in 14% of patients in both arms (2/14 per arm; 4/28 total). Early treatment dropout, defined as attending < 4 treatment sessions, occurred in 8% (1/13) of patients of the modified intent‐to‐treat sample; this patient discontinued SmartCBT after session 2, that is, after the cognitive preparation where patients are asked to make an informed decision for or against treatment. A total of 70% of SmartCBT patients (9/13) and 83% of CBT patients (10/12) attended at least half of the sessions (i.e., ≥ 8/16 sessions) and were considered treatment completers. Adherence was good with patients attending 11.2 ± 5.3 SmartCBT sessions (range 2–16) and 13.8 ± 3.9 CBT sessions (range 4–16) out of 16 sessions. Deviating from the study protocol, during the COVID‐19 pandemic, 4 patients each in the SmartCBT and CBT arms were offered online sessions (5.3 ± 2.4 SmartCBT sessions, range 2–7; 5.5 ± 2.4 CBT sessions, range 3–8). Assessment completion amounted to 62% (8/13) and 46% (6/13) at T1 and T2 in the SmartCBT arm and to 92% (11/12) and 75% (9/12) at the respective time points in the CBT arm (Figure 1), and was descriptively about 30% lower in the SmartCBT versus CBT arm.

As extracted from the trEATsmart logs, the app was used by 12/13 SmartCBT patients on 66% ± 29% of therapy days with 3 ± 2 entries per day (for the 12 app users: 72% ± 22% of therapy days with 3 ± 2 entries per day). Over the duration of therapy, the app delivered 37 ± 24 JITAIs, and patients planned to implement 92% ± 8% of these interventions. A total of 77% (10/13) of the SmartCBT patients used app‐based self‐monitoring of eating, 62% (8/13) used self‐monitoring of physical activity, 38% (5/13) used self‐monitoring of weight, and 46% (6/13) used the body image diary. To describe the overall app activity, 1467 messages (1341 reminders and 126 push notifications) were sent, on average 113 ± 119 messages per patient, including 103 ± 111 automated reminders and 10 ± 12 push notifications (for 12 app users: 122 ± 119 messages per patient, including 112 ± 111 automated reminders and 10 ± 12 push notifications).

**FIGURE 2:**
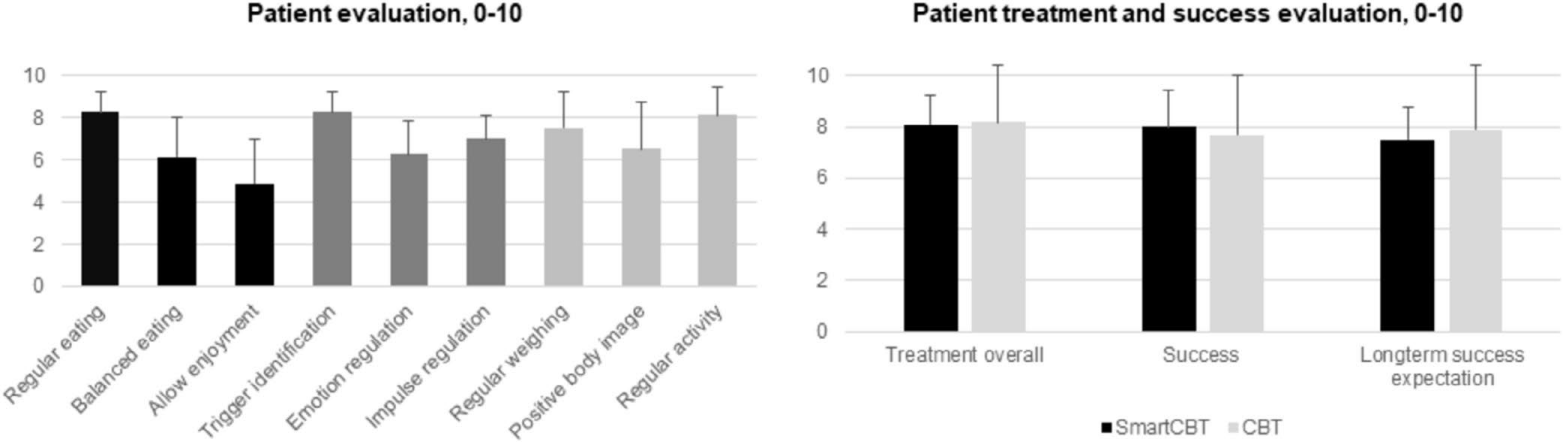
Acceptance ratings at posttreatment. Displayed are *M* and SD. Patient evaluation of the utility of the trEATsmart app was determined in the SmartCBT patients for whom a specific trEATsmart module was activated (Regular eating: 7/10, Balanced eating: 7/9, Allow enjoyment: 7/8, Trigger identification: 7/8, Emotion regulation: 7/9, Impulse regulation: 6/8, Positive body image: 4/5, Regular physical activity: 6/6, Regular weighing: 7/8). Patient evaluation of the treatment overall, success and long‐term success expectation was assessed in 6, 7, and 7 of 13 SmartCBT patients and in 9, 8, and 8 of 12 CBT patients. CBT, cognitive‐behavioral therapy; SmartCBT, smartphone‐supported CBT.

In the SmartCBT patients, the app yielded very good patient‐rated utility in the areas of regular eating, trigger identification, and regular physical activity (≥ 8, out of 10; Figure 2), and good utility in all other areas (6–7), except allowing enjoyment which was rated as moderately useful (4.9 ± 1.2). Patient‐rated usability of the trEATsmart app was favorable (8.3 ± 1.1, out of 10) as was the functionality, with problems with app use rarely reported (2.7 ± 1.8, rating 1–7; 7/12 patients with app use each). Patients' overall treatment evaluation at posttreatment was high in both arms (*d* = 0.0, 95% CI −1.0 to 0.9; Figure 2). With a small effect size, patients found SmartCBT to be more successful than CBT (*d* = 0.2, 95% CI −0.8 to 1.1), but expected lower long‐term treatment success (*d* = −0.2, 95% CI −1.1 to 0.7).

### 3.3 Efficacy Estimates

At T2, the number of OBEs decreased in SmartCBT and CBT from 24.9 and 11.6 to 3.5 and 2.9, respectively (Table 3). Thus, compared to CBT, the SmartCBT arm had a greater reduction of −12.7 OBEs at T2, indicating a large effect of *d* = −0.9 (95% CI, −2.0 to 0.1). However, when including gender as a covariate, this effect vanished (*β* = 0.0, 95% CI −6.9 to 6.9; Table 4).

**TABLE 3:** Raw data for the primary and secondary efficacy estimates (*N* = 25).

	Pretreatment (T0)		Midtreatment (T1)		Posttreatment (T2)		3‐month follow‐up (T3)	
	SmartCBT	CBT	SmartCBT	CBT	SmartCBT	CBT	SmartCBT	CBT
	*M* (SD)	*M* (SD)	*M* (SD)	*M* (SD)	*M* (SD)	*M* (SD)	*M* (SD)	*M* (SD)
Eating disorder symptoms								
Binge‐eating episodes (EDE)^a^	24.9 (13.4)	11.6 (10.8)	9.4 (8.9)	4.3 (5.9)	3.5 (3.2)	2.9 (5.8)	3.3 (3.8)	1.00 (2.2)
Eating disorder psychopathology (EDE‐Q)	3.7 (0.6)	3.3 (0.9)	3.1 (0.4)	2.9 (0.8)	2.7 (0.5)	2.1 (0.9)	2.7 (0.5)	1.9 (0.7)
General psychopathology								
Anxiety disorder symptoms (GAD‐7)	14.7 (4.3)	12.8 (4.2)	14.1 (4.7)	12.3 (4.0)	13.6 (4.6)	11.9 (3.9)	13.2 (3.7)	9.3 (2.2)
Self‐efficacy (GSES)	28.3 (4.5)	25.0 (5.2)	28.8 (6.5)	26.2 (5.5)	28.5 (3.3)	26.8 (6.3)	29.7 (5.4)	29.6 (5.0)
Emotion regulation (DERS)	88.4 (2.2)	87.3 (3.2)	81.0 (2.3)	85.2 (3.1)	73.1 (1.7)	75.3 (2.3)	71.5 (1.8)	65.6 (2.1)
Clinical impairment (CIA)	39.8 (9.1)	36.4 (8.2)	34.3 (13.6)	33.2 (8.2)	32.8 (12.5)	27.1 (6.0)	30.3 (10.9)	24.6 (5.9)
Well‐being								
Body mass index (kg/m^2^)	39.1 (9.0)	41.6 (8.7)	39.4 (8.7)	41.8 (9.3)	41.0 (8.6)	42.3 (9.2)	38.9 (7.5)	42.1 (8.7)
Waist‐to‐hip‐ratio	0.9 (0.1)	0.9 (0.1)	0.9 (0.1)	0.9 (0.1)	0.9 (0.1)	0.9 (0.1)	0.9 (0.1)	0.9 (0.1)
Quality of life (IWQoL‐Lite)	56.0 (20.3)	56.9 (19.9)	62.5 (19.5)	59.4 (15.4)	64.6 (22.6)	67.7 (18.5)	71.8 (21.8)	73.0 (23.8)

Abbreviations: CIA, Clinical Impairment Assessment (0–48*): DERS, Difficulties in Emotion Regulation Scale (36–180* less favorable scores are asterisked); EDE, Eating Disorder Examination; EDE‐Q, Eating Disorder Examination‐Questionnaire (0–6*); GAD‐7, Generalized Anxiety Disorder 7‐Item Scale (0–21*); GSES, General Self‐Efficacy Scale (10*–40); IWQoL‐Lite, Impact of Weight on Quality of Life‐Lite (0*–100).

^a^ Number of objective binge‐eating episodes over the past 28 days.

**TABLE 4:** Efficacy estimates in intent‐to‐treat analyses at posttreatment and 3‐month follow‐up: SmartCBT versus CBT (*N* = 25).

	SmartCBT versus CBT					
	Posttreatment (T2)		3‐month follow‐up (T3)		Time	
	*B* (95% CI)	*β* (95% CI)	*B* (95% CI)	*β* (95% CI)	*B* (95% CI)	*β* (95% CI)
Eating disorder symptoms						
Binge‐eating episodes (EDE)^a^	0.0 (−6.6 to 6.6)	0.0 (−6.9 to 6.9)	0.1 (−6.2 to 6.3)	0.0 (−6.5 to 6.6)	−3.4 (−5.2 to −1.6)	−3.7 (−5.5 to −1.9)
Eating disorder psychopathology (EDE‐Q)	0.0 (−0.5 to 0.5)	0.0 (−0.5 to 0.5)	0.0 (−0.5 to 0.5)	0.1 (−0.5 to 0.6)	−0.5 (−0.7 to −0.4)	−6.8 (−6.9 to −6.6)
General psychopathology						
Anxiety disorder symptoms (GAD‐7)	−0.2 (−2.5 to 2.2)	−0.1 (−2.6 to 2.3)	−0.1 (−2.3 to 2.1)	−0.1 (−2.4 to 2.2)	−1.1 (−1.8 to −0.4)	−3.1 (−3.8 to 2.4)
Self‐efficacy (GSES)	0.2 (−2.1 to 2.5)	0.2 (−2.3 to 2.6)	0.3 (−2.1 to 2.6)	0.2 (−2.2 to 2.7)	−1.6 (−2.3 to −0.9)	−4.4 (−5.1 to −3.7)
Emotion regulation (DERS)	0.0 (−1.6 to 1.6)	0.0 (−1.7 to 1.7)	0.0 (−1.5 to 1.5)	0.0 (−1.6 to 1.6)	−1.3 (−1.8 to −0.8)	−5.2 (−5.7 to −4.7)
Clinical impairment (CIA)	−0.2 (−4.7 to 4.3)	−0.1 (−4.8 to 4.6)	−0.1 (−4.3 to 4.1)	0.0 (−4.4 to 4.3)	−4.1 (−5.5 to −2.8)	−5.9 (−7.3 to −4.5)
Well‐being						
Body mass index (kg/m^2^)	−0.1 (−0.8 to 0.6)	−0.2 (−0.9 to 0.6)	−0.1 (−0.9 to 0.7)	−0.2 (−1.1 to 0.7)	0.2 (−0.1 to 0.4)	1.2 (0.9 to 1.4)
Waist‐to‐hip‐ratio	0.0 (0.0 to 0.0)	−0.1 (−0.2 to −0.1)	0.0 (0.0 to 0.0)	−0.2 (−0.2 to −0.1)	0.0 (0.0 to 0.0)	0.0 (−0.1 to 0.0)
Quality of life (IWQoL‐Lite)	0.3 (−6.5 to 7.0)	0.1 (−7.0 to 7.2)	0.0 (−9.8 to 9.9)	0.0 (−10.3 to 10.3)	−6.2 (−8.9 to −3.5)	−4.5 (−7.2 to −1.8)

*Note*: For interaction effects, a negative value indicates that SmartCBT is clinically better than CBT. For time effects across T0, T1, and T2, a negative sign indicates a clinical improvement.

Abbreviations: CBT, cognitive‐behavioral therapy; DERS, Difficulties in Emotion Regulation Scale; EDE, Eating Disorder Examination; EDE‐Q, Eating Disorder Examination‐Questionnaire; GAD‐7, Generalized Anxiety Disorder 7‐Item Scale; GSES, General Self‐Efficacy Scale; SmartCBT, smartphone‐supported CBT.

^a^ Number of objective binge‐eating episodes over the past 28 days.

Abstinence from binge eating at T2 and T3 occurred in 15% (2/13) and 23% (3/13) of SmartCBT patients and in 58% (7/12) of CBT patients at both timepoints (T2: NNT = −2.3, 95% CI −1.2 to 5.6; T3: NNT = −2.8, 95% CI −1.3 to 3.9). Remission from BED at T2 and T3 was achieved by 23% (3/13) and 15% (2/13) of SmartCBT patients, and 58% (7/12) and 67% (8/12) of CBT patients (T2: NNT = −2.8, 95% CI −1.3 to 3.9; T3: NNT = −2.0, 95% CI −1.2 to 9.2).

Regarding continuous measures, the SmartCBT group showed small‐sized advantages in BMI loss at T2 and T3 and waist‐to‐hip ratio reduction at T3, but lower improvement in self‐efficacy at T2 and T3 than the CBT group (Tables 3 and 4). All psychometric variables showed large decreases between T0 and T3, whereas BMI showed an increase overall.

### 3.4 Safety

In both arms, eight adverse events were recorded during therapy among eight patients, with no events judged to be attributable to treatment (Table S1). There were two serious adverse events in two patients unrelated to treatment (alcoholism relapse: SmartCBT; leg ulcer: CBT). Using a continuous measure of somatic complaints from T0 to T3, at most time points, the SmartCBT patients descriptively reported more severe, but less moderate or mild somatic complaints than the CBT patients (Table S2).

### 3.5 Target Engagement

Regarding target engagement for the normalization of eating behavior, regular eating, operationalized app‐based as intervals > 1 h and < 4 h between eating episodes (Table 1), was documented 1.9 ± 1.3 times per day with data entry and was associated with *r* = 0.1 (small effect) with greater OBE improvement at T2. Balanced eating in terms of a calorie‐moderated diet, operationalized as the amount of kcal consumed determined using app‐based eating self‐monitoring, amounted to 1447.7 ± 554.5 kcal intake per day, with lower kcal intake being correlated with greater OBE improvement at T2 with *r* = 0.3 (medium effect).

Target engagement for emotion regulation, assessed app‐based through the number of patients' reports of using emotion regulation skills during periods when the respective goal was activated (11.0 ± 10.2), was associated with a lower T2 OBE improvement (*r* = −0.1, small effect). In contrast, the number of patients' reports of using impulse regulation skills (35.7 ± 66.9) was associated with greater T2 OBE improvement (*r* = 0.3, medium effect).

Regarding target engagement for developing a positive body image, as assessed via the number of app‐based self‐monitoring records regarding body image (3.0 ± 1.8), physical activity (27.7 ± 14.9), and weight (7.3 ± 2.9) during periods when the respective goal was activated, was associated with a lower OBE improvement at T2 (*r* = −0.1, small effect; *r* = −0.2, small effect; *r* = −0.3, medium effect, respectively).

## 4 Discussion

This exploratory, assessor‐blind RCT aimed to assess the feasibility of CBT with adjunctive smartphone support and provide estimates for effects, safety, and target engagement using the trEATsmart app, adapted from the CBT+ app for bulimia nervosa (Juarascio et al. 2021, 2023), compared to CBT alone. The results support feasibility, including timely recruitment, low attrition, and high adherence (i.e., session attendance), consistent with previous research (de Zwaan et al. 2017; Hilbert et al. 2019). However, treatment completion was 13% higher in the CBT arm than in the SmartCBT arm, due to one SmartCBT patient discontinuing early. Assessment completion was high in the CBT arm, consistent with previous literature (de Zwaan et al. 2017), but it was moderate in SmartCBT, likely due to increased baseline psychopathology (see Vroling et al. 2016) and/or to the higher burden of app use requiring repeated entries, which differs from previous evidence in bulimia nervosa where the self‐monitoring requirement in CBT+ was less intense (Juarascio et al. 2023). Overall app use was substantial, with trEATsmart used most days of therapy, averaging three entries per day, and patients received a significant number of JITAIs, push notifications, and reminders, similar to CBT+ in bulimia nervosa (Juarascio et al. 2023).

Most patients utilized the app's self‐monitoring functions, especially for eating, but also for other areas. Patients rated the trEATsmart app's utility as very good or good in most areas, except for allow enjoyment, which was rated as moderately useful. This new module could be expanded to allow planning and realization of both pleasurable food‐ and nonfood‐related activities. Usability and functionality ratings of the trEATsmart app were positive, as previously reported for bulimia nervosa (Juarascio et al. 2023). While overall treatment evaluation was high in both arms, SmartCBT patients rated it as more successful but had lower expectations for long‐term success, with small effect sizes, presumably related to their increased psychopathological burden (Turner et al. 2019).

Regarding efficacy estimates, given this study's exploratory nature, significance testing was avoided, and efficacy estimates were interpreted with 95% CIs (Eldridge et al. 2016). These estimates must particularly be considered in light of the small sample size and notable baseline differences: despite randomization, SmartCBT patients had descriptively higher initial binge‐eating symptoms, comorbid anxiety, and lower education. Higher baseline binge eating and general psychopathology are known to negatively impact treatment response (Vall and Wade 2015), complicating attributions of differential outcomes to app support. For the primary efficacy estimate, OBEs were reduced more in SmartCBT than in CBT, with a large effect size. However, SmartCBT patients started with more than twice the number of monthly OBEs, increasing the potential for larger reductions (Hilbert et al. 2019); gender adjustment nullified this difference. Both abstinence from binge eating and remission from BED were achieved by about three times less patients in SmartCBT than CBT, with negative NNTs and potentially clinically significant improvements lower than typical CBT outcomes (Hilbert et al. 2019; Monteleone et al. 2022), likely related to elevated baseline binge eating. Both arms showed large reductions in continuous secondary efficacy estimates. With small effect sizes, SmartCBT showed greater reductions in BMI and waist‐to‐hip ratio, possibly related to the app's weight management skills focus, while self‐efficacy improvement appeared lower in SmartCBT, potentially due to baseline differences.

Safety was supported for both SmartCBT and CBT, with few (serious) adverse events unrelated to treatment, consistent with previous research (Hilbert et al. 2019). For target engagement, regular eating and eating a balanced, calorie‐moderated diet skills extracted from trEATsmart app logs were linked to posttreatment binge‐eating improvement with small‐to‐medium effects. Impulse regulation skill use was also positively associated with posttreatment binge‐eating reduction, while emotion regulation skills showed an inverse association. These findings align with D'Adamo et al. (2024), who examined prospective associations between skill use and binge eating during the same week or the following week in CBT+ augmented treatment of bulimia nervosa. Notably, the overall use of emotion regulation skills was low, possibly because this module was activated later in CBT. Self‐monitored energy intake likely reflects common underreporting, especially among those with higher weight (Howes et al. 2024). Unexpectedly, self‐monitoring of body image, physical activity, and weight was inversely associated with posttreatment binge‐eating improvement. It is possible that such self‐monitoring induces negative affect by raising awareness of negative aspects of body image, physical activity, or weight, although these modules received favorable evaluation (Figure 2).

With respect to strengths and limitations, this exploratory study followed key principles of RCT design for psychological treatment research to minimize risk of bias (e.g., minimal exclusion criteria, randomization, blinded assessments, modified intent‐to‐treat analysis, and reporting in accordance with the CONSORT Extension for randomized pilot and feasibility trials; Eldridge et al. 2016). However, the small sample size inherent to an exploratory RCT, differences in baseline psychopathology, and assessment noncompletion limit the generalizability. Additional variability may have resulted from a few necessary online sessions conducted with a small subset of patients during the COVID‐19 pandemic. Still, the results support the feasibility, safety, and target engagement, as well as changes over time in both treatment arms. While reliable sample size estimations cannot be derived from this study (Leon et al. 2011), design and methods may inform a future confirmatory RCT.

Further study design refinements should include self‐monitoring and skills‐monitoring in both SmartCBT and CBT to clarify the active ingredients of app support (e.g., self‐monitoring, digital augmentation) and to identify potential adverse effects. To improve assessment completion in the SmartCBT arm, patient‐requested features to reduce burden should be added (e.g., passive dietary self‐monitoring via barcode scanning, wearable sensor data import). In addition, positive reinforcement (e.g., visual feedback or encouraging messages following entries) should be strengthened to further support engagement. An assessment of nationality and origin should be included. For generalizability, the trEATsmart app, currently on Android—the most common operating system in Germany—should be adapted for iOS to ensure availability to all patients with BED. Assessing therapist‐perceived feasibility including acceptance is key for future dissemination if the app significantly enhances CBT effects. Evidence is further needed to understand the therapeutic process, acquisition and use of skills in relation to treatment outcome, and the effects of app support on the therapeutic alliance to optimize and avoid harm from digital augmentation. Designed to support change in skills, the app does not directly support change in cognitive schemata or processes underlying the maintenance of binge eating in BED. However, its potential to augment change in these areas merits further investigation.

In conclusion, in light of the growing evidence base on JITAIs in the treatment of diverse mental and physical health conditions (Hsu et al. 2025; Wang and Miller 2020), trEATsmart shows promise for BED treatment due to its timely mHealth approach, personalization, ease of use, and high acceptance. To overcome the limitations of this exploratory RCT, a larger confirmatory trial could clarify the adjunctive app's incremental efficacy, identify its mechanisms of action and potential moderators (e.g., self‐regulation; Presseller et al. 2022), and assess cost‐effectiveness.

## Author Contributions

**Anja Hilbert:** conceptualization, data curation, funding acquisition, investigation, methodology, project administration, resources, software, supervision, validation, visualization, writing – original draft, writing – review and editing. **Ulrike Klotz:** formal analysis, writing – original draft, writing – review and editing. **Sina Sadeghi:** formal analysis, writing – original draft, writing – review and editing. **Adrienne S. Juarascio:** conceptualization, software, validation, writing – original draft, writing – review and editing. **Toralf Kirsten:** formal analysis, writing – original draft, writing – review and editing.

## Ethics Statement

The authors assert that all procedures contributing to this work comply with the ethical standards of the relevant national and institutional committees on human experimentation and with the Helsinki Declaration of 1975, as revised in 2008.

## Conflicts of Interest

Dr. Hilbert reports receiving research grants from the German Federal Ministry of Education and Research, German Research Foundation, Innovation Fund, and Roland Ernst Foundation for Health Care; royalties for books on the treatment of eating disorders and obesity with Hogrefe and Kohlhammer; honoraria for workshops and lectures on eating disorders and obesity and their treatment, including for Lilly and Novo Nordisk; honoraria as associate editor of the *International Journal of Eating Disorders*; and honoraria as a consultant for Takeda. No other competing interests were reported.

## Supporting information


**Data S1.** Supporting Information.

## Data Availability

The data that support the findings of this study, materials, and code are available upon reasonable request from the corresponding author. The data are not publicly available due to privacy or ethical restrictions.
