# Supplementary material for: Smartphone‐Supported Cognitive‐Behavioral Therapy in Binge‐Eating Disorder: An Exploratory Randomized Trial
Source: Int J Eat Disord. 2025 Jun 16;58(9):1793–805. doi: 10.1002/eat.24479 (PMC12423581; doi:10.1002/eat.24479)
Supplement: Supplementary file 1 — Data S1. Supporting Information. [file EAT-58-1793-s001.docx]

**Online Supplementary Material for:**

Hilbert, A., Klotz, U., Sadeghi, S., Juarascio, A. S., & Kirsten, T.
Smartphone-Supported Cognitive-behavioral Therapy in Binge-Eating Disorder:
An Exploratory Randomized Trial

**eAppendix.** Supplementary Information

Supplementary Methods

**eTables.** Supplemental Tables

eTable 1. Number of adverse events verbally collected from patients at the outset of each session

eTable 2. Somatic complaints as assessed through the 15-item Patient Health Questionnaire (PHQ-15).

**Supplementary Methods**

**Measures: Psychometric self-report questionnaires**

Eating disorder psychopathology was assessed through the Eating Disorder Examination-Questionnaire (EDE-Q) (Fairburn & Beglin, 2008; Hilbert & Tuschen-Caffier, 2016b), the self-report version of the EDE. Beyond six diagnostic items, 22 items are assigned to the four subscales restraint, eating concern, weight concern, and shape concern, and provided with a 7-point Likert scale ranging from 0=*feature absent* to 6=*feature present every day* or *to an extreme degree*. From the subscale mean scores, a global mean score was calculated, with higher values of greater eating disorder psychopathology, and acceptable internal consistency based on this study’s data (Cronbach’s α =.79, 95% CI .64 to .93; McDonald’s ω =.81, 95% CI .64 to .91). The subscale scores were not reported because of Cronbach’s α <.70 for the weight concern subscale.

Anxiety disorder symptoms were collected using the Generalized Anxiety Disorder 7 (GAD-7) (Löwe et al., 2008; Spitzer et al., 2006), rated on a 4-point Likert scale for the last two weeks, with a range from 0=*not at all* to 3=*almost every day*. A higher global sum score (0–21) indicates more severe generalized anxiety symptoms, and a score ≥15 was applied to determine probable cases of moderate generalized anxiety disorder (α=.82, 95% CI .72 to .93; ω=.83, 95% CI .71 to .92).

Depressive symptoms were measured using the 9-item Patient Health Questionnaire (PHQ-D) (Gräfe et al., 2004; Spitzer et al., 1999), assessing these symptoms over the last two weeks on a 4-point Likert scale ranging from 0=*not at all* to 3=*almost every day*. A higher global sum score (0–27) indicates more severe depressive symptoms, with a score ≥ 10 indicating probable cases for moderate depression. However, with Cronbach’s α <.70, the global sum score was not reported as an outcome measure. Instead, we the used the dichotomous score of moderate depression for sample description at baseline.

Global self-efficacy in coping with challenging situations was measured using the 10-item General Self-Efficacy Scale (GSES) (Schwarzer & Jerusalem, 1995). Patients were asked to display their own competence to cope with such situations on a 4-point Likert scale ranging from 1=*not at all true* to 4=*exactly true*. Higher total sum scores indicate greater general self-efficacy (α=.88, 95% CI .81 to .94; ω=.89, 95% CI .81 to .94).

Emotion regulation deficits were assessed through the Difficulties in Emotion Regulation Scale (DERS) (Ehring et al., 2008; Gratz & Roemer, 2004). The 36 items were supplied with a 5-point Likert scale ranging from 1=*almost never* *(0%*–*10%)* to 5=*almost always (91%*–*100%)*. Higher total sum scores (36–180) indicate greater deficits in emotion regulation (α=.94, 95% CI .91 to .97; ω=.89, 95% CI .82 to .94).

Impairment due to eating disorder psychopathology was measured through the Clinical Impairment Assessment (CIA) (Bohn et al., 2008; de Zwaan et al., 2017). The 16 items of the CIA are rated from 0=*not at all* to 3=*a lot* and a sum score is calculated, with higher scores indicating a greater level of impairment (α=.86, 95% CI .79 to .94, ω=.87, 95% CI .78 to .93).

Weight-related quality of life was measured using the Impact of Weight on Quality of Life–Lite (IWQOL-Lite) (Kolotkin et al., 2001; Mueller et al., 2011)*.* Its 31 items covering five domains (physical function, self-esteem, sexual life, public distress, work) were rated on a 5-point Likert scale from 1=*never true* to 5=*always true* for the last week. The IWQOL-Lite total sum score was computed and standardized to 0-100, with higher scores indicating better health-related quality of life (α=.93, 95% CI .89 to .97, ω=.93, 95% CI .89 to .97).

Regarding safety, adverse events were assessed verbally prior to each session by the therapist. In addition, the 15-item Patient Health Questionnaire (PHQ-15) (Gräfe et al., 2004; Spitzer et al., 1999) was used to have patients rate somatic symptoms over the past four weeks on a 3-point Likert scale ranging from 0=*not bothered* to 2=*bothered a lot* at T0-T3. A higher global sum score indicates more severe symptoms (α=.79, 95% CI .68 to .90; ω not determined because of variables with consistent zero ratings).

**References**

Bohn, K., Doll, H. A., Cooper, Z., O'Connor, M., Palmer, R. L., Fairburn, C. G. (2008). The measurement of impairment due to eating disorder psychopathology. *Behaviour Research and Therapy, 46,* 1105–1110. <https://doi.org/10.1016/j.brat.2008.06.012>

de Zwaan, M., Herpertz, S., Zipfel, S., Svaldi, J., Friederich, H. C., Schmidt, F., Mayr, A., Lam, T., Schade-Brittinger, C., & Hilbert, A. (2017). Effect of Internet-based guided self-help vs individual face-to-face treatment on full or subsyndromal binge eating disorder in overweight or obese patients: The INTERBED randomized clinical trial. *JAMA Psychiatry*, *74*, 987–995. https://doi.org/ 10.1001/jamapsychiatry.2017.2150

Ehring, T., Fischer, S., Schnülle, J., Bösterling, A., & Tuschen-Caffier, B. (2008). Characteristics of emotion regulation in recovered depressed versus never depressed individuals. *Personality and Individual Differences, 44,* 1574–1584. <https://doi.org/10.1016/j.paid.2008.01.013>

Fairburn, C. G. & Beglin, S. J. (2008). *Eating Disorder Examination-Questionnaire (Edition 6.0)*. https://www.credo-oxford.com/pdfs/EDE-Q_6.0.pdf

Gräfe, K., Zipfel, S., Herzog, W., & Löwe, B. (2004). Screening psychischer Störungen mit dem Gesundheitsfragebogen für Patienten (PHQ-D). *Diagnostica, 50,* 171–181. https://doi.org/ 10.1026/0012-1924.50.4.171

Gratz, K. L. & Roemer, L. (2004). Multidimensional assessment of emotion regulation and dysregulation: Development, factor structure, and initial validation of the difficulties in emotion regulation scale. *Journal of Psychopathology and Behavioral Assessment, 26,* 41–54. https://doi.org/10.1023/ B:JOBA.0000007455.08539.94

Hilbert, A. & Tuschen-Caffier, B. (2016b). *Eating Disorder Examination-Questionnaire: Deutsch­sprachige Übersetzung [Eating Disorder Examination-Questionnaire: German trans­lation]*, 2nd ed. dgvt-Verlag.

Juarascio, A. S., Presseller, E. K., Srivastava, P., Manasse, S. M., & Forman, E. M. (2023). A randomized controlled trial of CBT+: A clinician-controlled, just-in-time, Adjunctive intervention for bulimia-spectrum disorders. *Behavior Modification, 47,* 551–572. https://doi.org/10.1177/ 01454455221109434

Juarascio, A., Srivastava, P., Presseller, E., Clark, K., Manasse, S., & Forman, E. (2021). A clinician-controlled just-in-time adaptive intervention system (CBT+) designed to promote acquisition and utilization of cognitive behavioral therapy skills in bulimia nervosa: Development and preliminary evaluation study. *JMIR Formative Research, 5,* e18261. <https://doi.org/10.2196/18261>

Kolotkin, R. L., Crosby, R. D., Kosloski, K. D., & Williams, G. R. (2001). Development of a brief measure to assess quality of life in obesity. *Obesity Research, 9,* 102–111. https://doi.org/10.1038/ oby.2001.13

Löwe, B., Decker, O., Müller, S., Brähler, E., Schellberg, D., Herzog, W., & Herzberg, P. Y. (2008). Validation and standardization of the Generalized Anxiety Disorder Screener (GAD-7) in the general population. *Medical Care, 46*, 266–274. <https://doi.org/10.1097/MLR.0b013e318160d093>

Mueller, A., Holzapfel, C., Hauner, H., Crosby, R. D., Engel, S. G., Mühlhans, B., Kolotkin, R. L., Mitchell, J. E., Horbach, T., & de Zwaan, M. (2011). Psychometric evaluation of the German version of the impact of weight on Quality of Life-Lite (IWQOL-Lite) questionnaire. *Experimental and Clinical Endocrinology & Diabetes*, *119*, 69–74. https://doi.org/10.1055/s-0030-1261922

Schwarzer, R. & Jerusalem, M. (1995). Generalized Self-Efficacy scale. In J. Weinman, S. Wright, & M. Johnston (Eds.), *Measures in Health Psychology: A User’s Portfolio* (pp. 35–37). NFER-Nelson.

Spitzer, R. L., Kroenke, K., & Williams, J. B. (1999). Validation and utility of a self-report version of PRIME-MD: the PHQ primary care study. Primary care evaluation of mental disorders. Patient health questionnaire. *JAMA, 282,* 1737–1744. https://doi.org/10.1001/ jama.282.18.1737

Spitzer, R. L., Kroenke, K., Williams, J. B. W., & Löwe, B. (2006). A brief measure for assessing generalized anxiety disorder: the GAD-7. *Archives of Internal Medicine, 166,* 1092–1097. <https://doi.org/10.1001/archinte.166.10.1092>

**eTable 1. Number of adverse events verbally collected from patients at the outset of each session.**

|  | **SmartCBT**  **(*n*=13)** | **CBT**  **(*n*=12)** |
| --- | --- | --- |
|  | **No. (%)** | **No. (%)** |
| Adverse events | 5 (38%) | 3 (25%) |
| Patients with adverse events | 5 (38%) | 3 (25%) |
| Severity of adverse events |  |  |
| Mild | 1(20%) | 1(33%) |
| Moderate | 2 (40%) | 2 (66%) |
| Severe | 2 (40%) | 1 (33%) |
| Number of serious adverse events | 1 (20%) | 1 (33%) |
| Causal relationship to intervention |  |  |
| No | 5 (100%) | 4 (100%) |
| Possibly | 0 | 0 |
| Yes | 0 | 0 |

*Notes.* CBT, cognitive-behavioral therapy; SmartCBT, smartphone-supported CBT.

**eTable 2. Somatic complaints as assessed through the 15-item Patient Health Questionnaire (PHQ-15).**

| **Time point** | **Arm** | **N valid cases** | **Severity of somatic complaints** | | | |
| --- | --- | --- | --- | --- | --- | --- |
|  |  |  | Minimal < 5 | Mild ≥ 5 | Moderate ≥ 10 | Severe ≥ 15 |
| **T0** | SmartCBT | 13 | 2 (15.38%) | 4 (30.77%) | 3 (23.08%) | 4 (30.77%) |
|  | CBT | 12 | 1 (8.33%) | 4 (33.33%) | 4 (33.33%) | 3 (25.00%) |
| **T1** | SmartCBT | 9 | 2 (22.22%) | 1 (11.11%) | 2 (22.22%) | 4 (44.44%) |
|  | CBT | 10 | 1 (10.00%) | 4 (40.00%) | 1 (10.00%) | 4 (40.00%) |
| **T2** | SmartCBT | 8 | 3 (37.50%) | 1 (12.50%) | 0 | 4 (50.00%) |
|  | CBT | 10 | 1 (10.00%) | 3 (30.00%) | 5 (50.00%) | 1 (10.00%) |
| **T3** | SmartCBT | 5 | 1 (20.00%) | 1 (20.00%) | 0 | 3 (60.00%) |
|  | CBT | 6 | 2 (33.33%) | 2 (33.33%) | 2 (33.33%) | 0 |

*Notes.* CBT, cognitive-behavioral therapy; SmartCBT, smartphone-supported CBT.
